# Diagnosis of SLAP lesions on shoulder MRI using a 2.5D deep learning and ensemble learning framework

**DOI:** 10.3389/fsurg.2026.1730726

**Published:** 2026-03-05

**Authors:** Hongyu Wang, Qingyun Xue, Lei Shi, Fei Wang, Guanghan Gao, Lin Wang

**Affiliations:** 1 Department of Orthopaedics, Beijing Hospital, National Center of Gerontology, Institute of Geriatric Medicine, Chinese Academy of Medical Sciences, Beijing, China; 2 Peking University Fifth School of Clinical Medicine, Beijing, China

**Keywords:** artificial intelligence, deep learning, diagnostic model, ensemble learning, shoulder MRI, SLAP lesion

## Abstract

**Background:**

Superior labrum anterior and posterior (SLAP) lesions are a common cause of shoulder pain and instability. Developing accurate, non-invasive diagnostic tools is essential to support clinical decision-making for SLAP lesions. This study aimed to establish an automated diagnostic model for SLAP lesions using a 2.5D deep learning framework combined with ensemble learning and to evaluate its clinical utility.

**Methods:**

In this retrospective study, 185 patients who underwent shoulder arthroscopy between January 2019 and September 2025 were included (91 SLAP lesions, 94 controls). Preoperative shoulder magnetic resonance imaging (MRI) data were analysed. Images from three consecutive slices, centred on the maximal region of interest (ROI), were processed using a Wide_ResNet101_2 network pre-trained on ImageNet for deep feature extraction and probability prediction. A decision-level fusion strategy integrated the predicted probabilities from all three layers as input features for three ensemble classifiers: AdaBoost, Random Forest, and XGBoost. Model performance was assessed with accuracy, area under the receiver operating characteristic curve (AUC), sensitivity, specificity, precision, and F1-score. The DeLong test and integrated discrimination improvement (IDI) were used to compare models.

**Results:**

All ensemble models exhibited robust diagnostic performance. On the test set, the XGBoost model achieved the highest AUC (0.754) and sensitivity (0.933), though specificity was moderate (0.538). The Random Forest model yielded an AUC of 0.745, while the AdaBoost model achieved an AUC of 0.731. F1-scores ranged from 0.75 to 0.80. There were no statistically significant differences in AUC among the models. Feature importance analysis highlighted the central MRI slice as most contributory. Model interpretability assessments showed that the network focused predominantly on the biceps-labral complex, which is anatomically consistent with SLAP pathology.

**Conclusions:**

The proposed automated diagnostic model, utilising a 2.5D deep learning and ensemble approach, demonstrated favourable diagnostic performance and clinical applicability for SLAP lesion detection on shoulder MRI. Among the ensemble strategies, the XGBoost model provided the highest sensitivity, rendering it particularly suitable as a clinical decision-support tool. The multi-slice information fusion framework substantially improved diagnostic accuracy, supporting its potential as a novel artificial intelligence solution to assist radiologists in diagnosing shoulder labral injuries.

## 1 Introduction

Superior labrum from anterior to posterior (SLAP) lesions are among the most common shoulder injuries encountered in clinical practice and represent a major cause of shoulder pain and functional impairment (1). With the increasing popularity of sports and the accelerating pace of population ageing, SLAP lesions are attracting growing attention in clinical settings. According to Zhang et al. (2), the number of arthroscopic SLAP repairs in the United States increased by 105% between 2004 and 2009. Precise identification of SLAP lesions is essential to inform appropriate therapeutic strategies, avoid unnecessary surgical interventions, and minimise the healthcare burden.

However, SLAP lesions often present with non-specific symptoms and no single physical examination test is diagnostic. In routine practice, surgeons commonly use physical tests such as the O'Brien test, the Speed test, the anterior slide test, and the crank test. However, these tests have variable diagnostic accuracy and are influenced by operator experience and technique. Furthermore, they show substantial overlap with other shoulder disorders, making SLAP lesions difficult to confirm on clinical examination alone (3, 4). As a result, imaging modalities, particularly magnetic resonance imaging (MRI), have become indispensable adjuncts in the diagnostic evaluation of SLAP lesions (5–7). Nevertheless, recent meta-analyses indicate that conventional MRI has only moderate sensitivity (63%) and relatively high specificity (87.2%) for SLAP detection, suggesting that diagnostic accuracy can be further improved (8). Magnetic resonance arthrography (MRA) has demonstrated superior sensitivity and specificity (0.86 and 0.91, respectively) (9), but its invasive nature and associated infection risk limit its widespread use in clinical practice.

Amid these challenges, artificial intelligence (AI)-driven approaches show considerable promise in musculoskeletal imaging. Radiomics, which enables high-throughput extraction of quantitative imaging features combined with advanced machine learning algorithms, has advanced the field from qualitative assessment to quantitative, precision diagnostics (10, 11). More recently, deep learning, a subfield of machine learning, has outperformed traditional radiomics by enabling automated extraction and hierarchical learning of imaging features without hand-crafted feature engineering (12–17). Deep learning-based models have achieved strong performance in diagnosing fractures, assessing arthritis, and identifying spinal disorders, demonstrating transformative potential across orthopaedic imaging (18–21). In deep learning-based diagnosis of shoulder disorders, researchers have made valuable progress. For instance, Yao et al. (22) reported sensitivity and specificity of 85% using a custom convolutional neural network (CNN) for supraspinatus tear diagnosis, while Ni et al. (23) achieved area under the receiver operating characteristic (ROC) curve (AUC) values exceeding 0.95 for automated grading of partial-thickness rotator cuff tears on multisequence MRI. Guo et al. (24) utilised a CNN based on the Xception architecture to diagnose supraspinatus tendon tears, using arthroscopy as the gold standard. Their results showed that the model achieved diagnostic accuracy exceeding that of junior clinicians and comparable to that of senior clinicians.

Currently, studies on deep learning-based intelligent diagnosis of SLAP lesions remain limited, and the performance of existing models requires further validation (25). This study aims to establish an automated deep learning model for diagnosing SLAP lesions, providing a scientific and technical foundation for accurate diagnosis.

## 2 Materials and methods

### 2.1 Study design and ethical approval

This retrospective study was approved by the Ethics Committee of Beijing Hospital (approval No. 2025BJYYEC-KY341-01), and the requirement for informed consent was waived. All procedures were conducted in accordance with the ethical principles outlined in the Declaration of Helsinki.

### 2.2 Study population and inclusion/exclusion criteria

Clinical and imaging data were consecutively collected from patients who underwent shoulder arthroscopy for shoulder disorders at our institution between January 2019 and September 2025. Demographic characteristics, including age, sex, duration of symptoms, and history of trauma, were recorded for statistical analysis. Inclusion criteria were: (1) patients who underwent shoulder arthroscopy; and (2) completion of preoperative MRI examinations. Exclusion criteria were: (1) a history of previous shoulder surgery; (2) a history of severe shoulder trauma, shoulder tumours, or infectious diseases; (3) incomplete or poor-quality MRI examinations unsuitable for diagnostic assessment; and (4) absence of essential clinical data.

**Figure 1:**
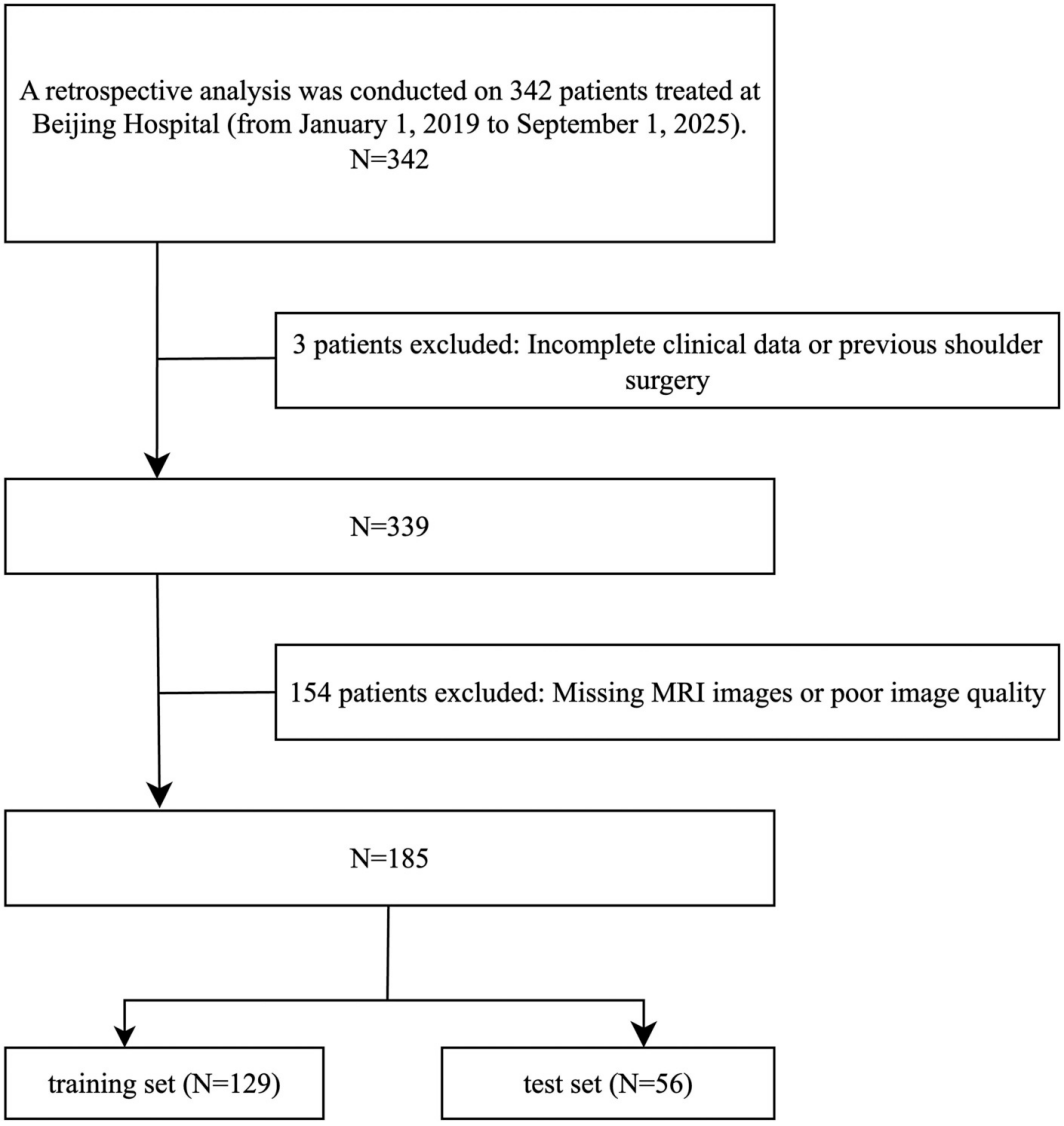
Flow diagram of subject enrollment.

Eligible patients were randomly assigned to the training and testing cohorts in a 7:3 ratio. This split was selected to balance the need for adequate data to support model training and hyperparameter optimisation with the need to retain a sufficiently sized, fully independent test set to yield robust estimates of diagnostic performance. Given the modest sample size and the aim to report performance on a truly independent cohort, we prioritised a hold-out test strategy rather than *k*-fold cross-validation, as cross-validation would reuse cases for both training and validation and could lead to optimistic performance estimates. A detailed flowchart is presented in Figure 1. The resulting sample size is comparable to previously published diagnostic deep learning studies in shoulder MRI cohorts, which commonly used similar single-centre sample sizes (22, 26, 27).

### 2.3 MRI protocol and arthroscopic reference standard

All patients underwent standardised non-contrast shoulder MRI on one of two 3.0T MRI scanners. The standardised scanning protocol comprised multiple sequences. In this study, images from the oblique coronal proton density-weighted fat-suppressed (PD FS) and oblique coronal T2-weighted fat-suppressed (T2WI FS) sequence were analysed. Images were acquired with standardised soft-tissue window settings and exported in Digital Imaging and Communications in Medicine (DICOM) format. All arthroscopic procedures were performed by experienced orthopaedic surgeons. The intra-articular structures, including the glenohumeral joint, the long head of the biceps tendon, and the biceps-labral complex, were systematically examined through standard posterior, anterior, and anterolateral portals. The integrity, continuity, and glenoid attachment of the superior labrum were carefully assessed. A definitive diagnosis of SLAP lesion was made intraoperatively if a superior labral tear or detachment of the biceps anchor from the glenoid was observed. Rotator cuff pathology was also documented and categorised as no tear, partial-thickness tear, or full-thickness tear.

### 2.4 Image preprocessing and region of interest (ROI) annotation

All MRI DICOM data were converted to NIfTI format for subsequent analysis. Volumes were resampled to isotropic 1 mm voxels, and window width/level normalisation was applied to ensure standardised contrast and brightness. ROI annotation was performed independently by two orthopaedic surgeons, each with over five years of experience in shoulder imaging interpretation. Discrepancies were resolved by consensus discussion. To assess inter-rater reliability, the intraclass correlation coefficient (ICC) was calculated for ROI segmentation agreement between the two orthopaedic surgeons. ICC values ranged from 0.832 to 0.983 [median = 0.956, interquartile range (IQR): 0.929–0.970], indicating high inter-rater agreement. The ITK-SNAP software (28) was used for manual delineation of the superior labrum on oblique coronal fat-suppressed sequences, including PD FS and T2WI FS. ROIs were segmented within the anatomical boundaries of the superior labrum on three to five consecutive slices to ensure three-dimensional anatomical coverage. The final multi-slice ROI for each patient was saved as a single ROI file. In total, MRI scans from 185 subjects were annotated, and all annotations underwent duplicate review and quality assurance.

### 2.5 2.5D deep learning modelling strategy

A 2.5D modelling strategy was implemented. For each subject, the central slice containing the largest ROI was identified, and this slice, together with its immediately superior and inferior adjacent slices, was analysed as a three-slice set. Each slice was cropped to a bounding box containing the largest ROI. Data augmentation techniques, including random horizontal flipping, vertical flipping, and cropping, were applied to enhance data diversity. All images were resized to 224 × 224 pixels to ensure compatibility with the network input.

**Figure 2:**
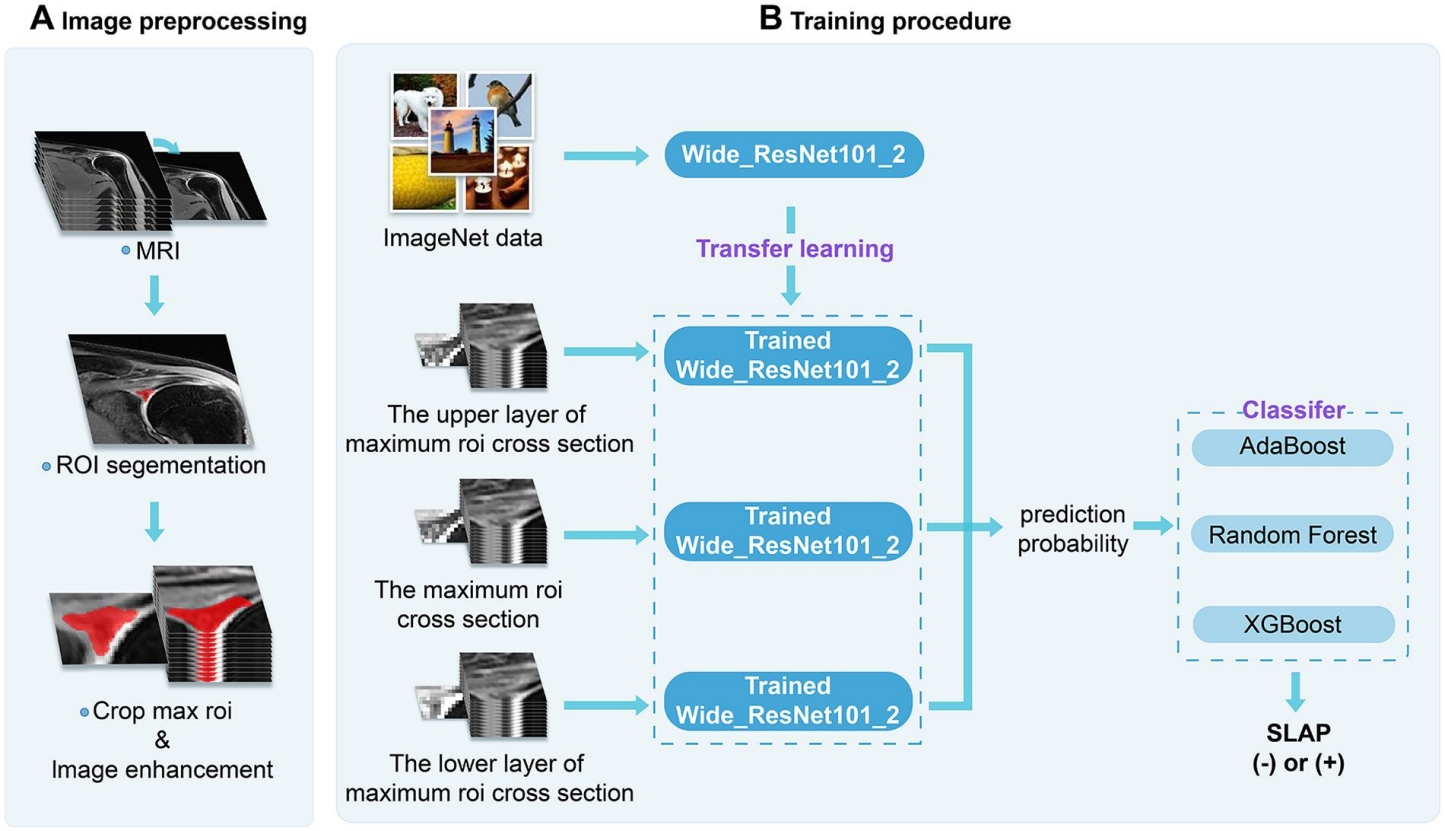
Schematic illustration of the image preprocessing and model training pipeline. The left panel **(A)** depicts the image preprocessing workflow, including MRI DICOM data input, ROI segmentation on oblique coronal T2-weighted fat-suppressed images, and image enhancement procedures. The right panel **(B)** illustrates the model training procedure, comprising transfer learning with the Wide_ResNet101_2 backbone, parallel extraction of diagnostic probabilities from three ROI cross-sections (centre, upper, and lower slices), and final binary SLAP classification using AdaBoost, Random Forest, and XGBoost ensemble classifiers.

The Wide_Resnet101_2 network, a modified version of ResNet with increased network width to improve feature representation (29), was selected as the backbone for deep feature extraction. The network was initialised with ImageNet-pretrained weights and subsequently fine-tuned on the shoulder MRI dataset to adapt to domain-specific features. Training was conducted on a workstation equipped with an Intel i9-14900KF CPU, an NVIDIA GeForce RTX 4070Ti Super GPU, and 64 GB RAM, using PyTorch version 1.8.0. Training parameters were as follows: batch size 32; 40 epochs; optimiser: Stochastic Gradient Descent (SGD); initial learning rate 0.01; image pre-processing using ImageNet standard normalisation. For each subject, the three cropped slices were input simultaneously into three parallel Wide_Resnet101_2 subnetworks. Each subnetwork processed one slice and output a diagnostic probability. The three probabilities corresponding to the centre, upper, and lower slices were then combined through a decision-level fusion mechanism to yield the final SLAP diagnosis. A schematic illustration of the image preprocessing and model training workflow is provided in Figure 2.

### 2.6 Ensemble classifier construction and decision fusion

To fully leverage the multi-planar (2.5D) information, a late-fusion decision-level strategy was adopted. The independently predicted probabilities from the three slices (P_centre, P_upper, P_lower) generated by the respective subnetworks, were combined to form the feature vector F = [P_centre, P_upper, P_lower]^T. This feature vector was input into three different ensemble classifiers within a stacking ensemble framework: AdaBoost, Random Forest, and XGBoost (30, 31). Each meta-classifier learned the non-linear relationships and optimal weighting among slice-level predictions and produced the final binary SLAP diagnosis. The dataset was divided into a training set (*n* = 129) and a test set (*n* = 56), consistent with the deep learning model. The training set was used for model training and hyperparameter tuning, while the test set was reserved for independent validation.

### 2.7 Model performance evaluation and statistical analysis

The diagnostic performance of the XGBoost, Random Forest, and AdaBoost ensembles was evaluated on the training and test sets. Metrics included accuracy, AUC, sensitivity, specificity, precision, and F1-score. Comparative performance across models was assessed using the DeLong test for AUCs (32). Integrated discrimination improvement (IDI) was calculated to quantify reclassification improvement between models. Pairwise comparison matrices were established to examine relative model performance (33).

All analyses were conducted in Python 3.7 using PyTorch 1.8.0 (for deep learning) and scikit-learn (for ensemble learning). Normality of continuous variables was assessed using the Shapiro–Wilk test. As all continuous variables deviated from normality, they are presented as median (IQR), and between-group comparisons were performed using the Mann–Whitney *U*-test. Categorical variables are presented as count (percentage) and compared using the chi-square test or Fisher's exact test, as appropriate. A two-sided *p*-value < 0.05 was considered statistically significant.

## 3 Results

### 3.1 Baseline characteristics and data distribution

A total of 185 patients undergoing shoulder arthroscopy were included in this study. Among them, 91 patients (49.2%) were diagnosed intraoperatively with SLAP lesions, while 94 patients (50.8%) showed no evidence of SLAP lesions (no-lesion group). There were no statistically significant differences between the SLAP and no-lesion groups in age, sex, symptom duration, or history of trauma (all *p* > 0.05). Patients were randomly assigned to the training set (*n* = 129; 61 with SLAP lesions, 68 without) and the independent test set (*n* = 56; 30 with SLAP lesions, 26 without) in a 7:3 ratio. Further clinical characteristics are detailed in Table 1.

**Table 1:** Demographic and clinical characteristics of patients in training and test sets.

Characteristics	Entire cohort	Test set	Training set	*p* value
Age (years), median (IQR)	63 (57–69)	63.5 (58.25–69)	63 (57–69)	0.926
Gender				0.509
Man	114 (61.622)	32 (57.143)	82 (63.566)	
Woman	71 (38.378)	24 (42.857)	47 (36.434)	
History of trauma, *n* (%)				0.336
No	104 (56.216)	28 (50.000)	76 (58.915)	
Yes	81 (43.784)	28 (50.000)	53 (41.085)	
Symptom duration (months), median (IQR)	6 (3–12)	6 (4–12)	6 (2–11.5)	0.347
Arthroscopic full-thickness rotator cuff tear, *n* (%)				0.06
No	97 (52.432)	23 (41.071)	74 (57.364)	
Yes	88 (47.568)	33 (58.929)	55(42.636)	

### 3.2 Model interpretability

**Figure 3:**
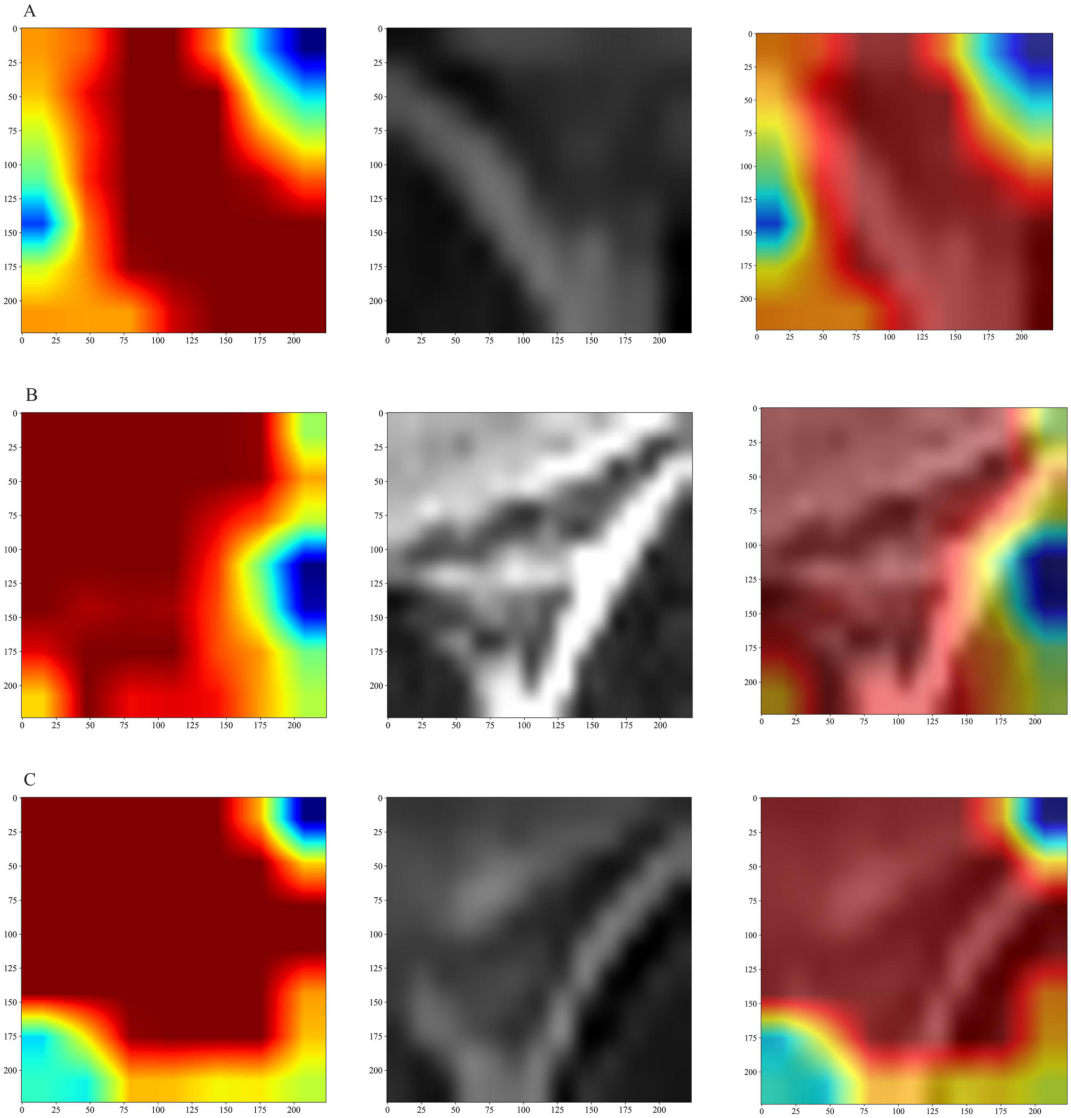
Grad-CAM visualization of deep learning model predictions. Grad-CAM visualizations of deep learning model predictions for three different imaging cases, labeled as **(A–C)**. For each case, images from left to right represent: (1) the raw heatmap generated by Grad-CAM, (2) the maximal bounding rectangle of the region of interest (ROI), and (3) an overlay of the heatmap with the bounding rectangle. Grad-CAM generates a “heatmap” to highlight the regions in the image that are most critical for the model's decision.

The diagnostic decision patterns of the deep learning model were analysed using Gradient-weighted Class Activation Mapping (Grad-CAM) (34). Visualisations showed that the model consistently focused on the superior labrum–biceps anchor complex, a region anatomically relevant to SLAP pathology. The resulting heatmaps demonstrated the model's capacity to localise lesion sites, providing transparent and intuitive decision support for surgeons (Figure 3).

### 3.3 Diagnostic performance of the model

**Figure 4:**
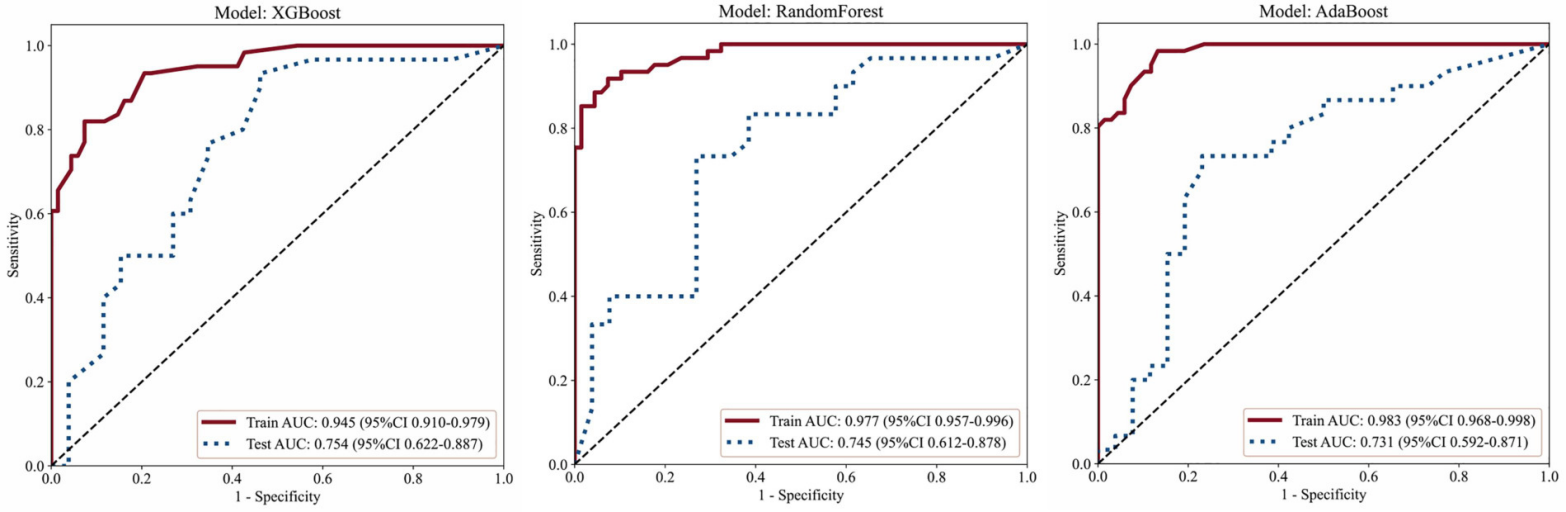
ROC curves of the three machine learning models evaluated on the training and testing datasets.

Within the training set, the Random Forest model achieved the highest fitting accuracy [accuracy = 0.922, AUC = 0.977 (95% CI: 0.957–0.996)], indicating strong internal learning performance. On the independent test set, the XGBoost model achieved the highest AUC at 0.754 (95% CI: 0.622–0.887), slightly outperforming Random Forest [AUC = 0.745 (95% CI: 0.612–0.878)] and AdaBoost [AUC = 0.731 (95% CI: 0.592–0.871)]. The XGBoost model also demonstrated superior sensitivity [0.933 (95% CI: 0.779–0.992)], albeit with lower specificity [0.538 (95% CI: 0.334–0.734)]. Across all three ensemble models, F1-scores on the test set ranged from 0.75 to 0.80, indicating reliable diagnostic consistency. Both Random Forest and AdaBoost maintained sensitivity and specificity ≥0.70 on the test set, and classification accuracy for all models exceeded 0.70, underscoring robust diagnostic performance. Comprehensive metrics and ROC curves are presented in Table 2 and Figure 4.

**Table 2:** Diagnostic performance of three ensemble machine learning models for SLAP lesion detection.

Model	Dataset	AUC (95%CI)	Sensitivity (95%CI)	Specificity (95%CI)	Accuracy	Precision	F1
XGBoost	Training set	0.945 (0.910–0.979)	0.820 (0.700–0.906)	0.926 (0.837–0.976)	0.876	0.909	0.862
	Test set	0.754 (0.622–0.887)	0.933 (0.779–0.992)	0.538 (0.334–0.734)	0.750	0.700	0.800
RandomForest	Training set	0.977 (0.957–0.996)	0.967 (0.887–0.996)	0.441 (0.321–0.567)	0.922	0.918	0.918
	Test set	0.745 (0.612–0.878)	0.700 (0.506–0.853)	0.731 (0.522–0.884)	0.732	0.759	0.746
AdaBoost	Training set	0.983 (0.968–0.998)	0.984 (0.912–0.999)	0.868 (0.764–0.938)	0.853	0.776	0.861
	Test set	0.731 (0.592–0.871)	0.700 (0.506–0.853)	0.769 (0.564–0.910)	0.714	0.750	0.724

### 3.4 Statistical comparison between models

**Figure 5:**
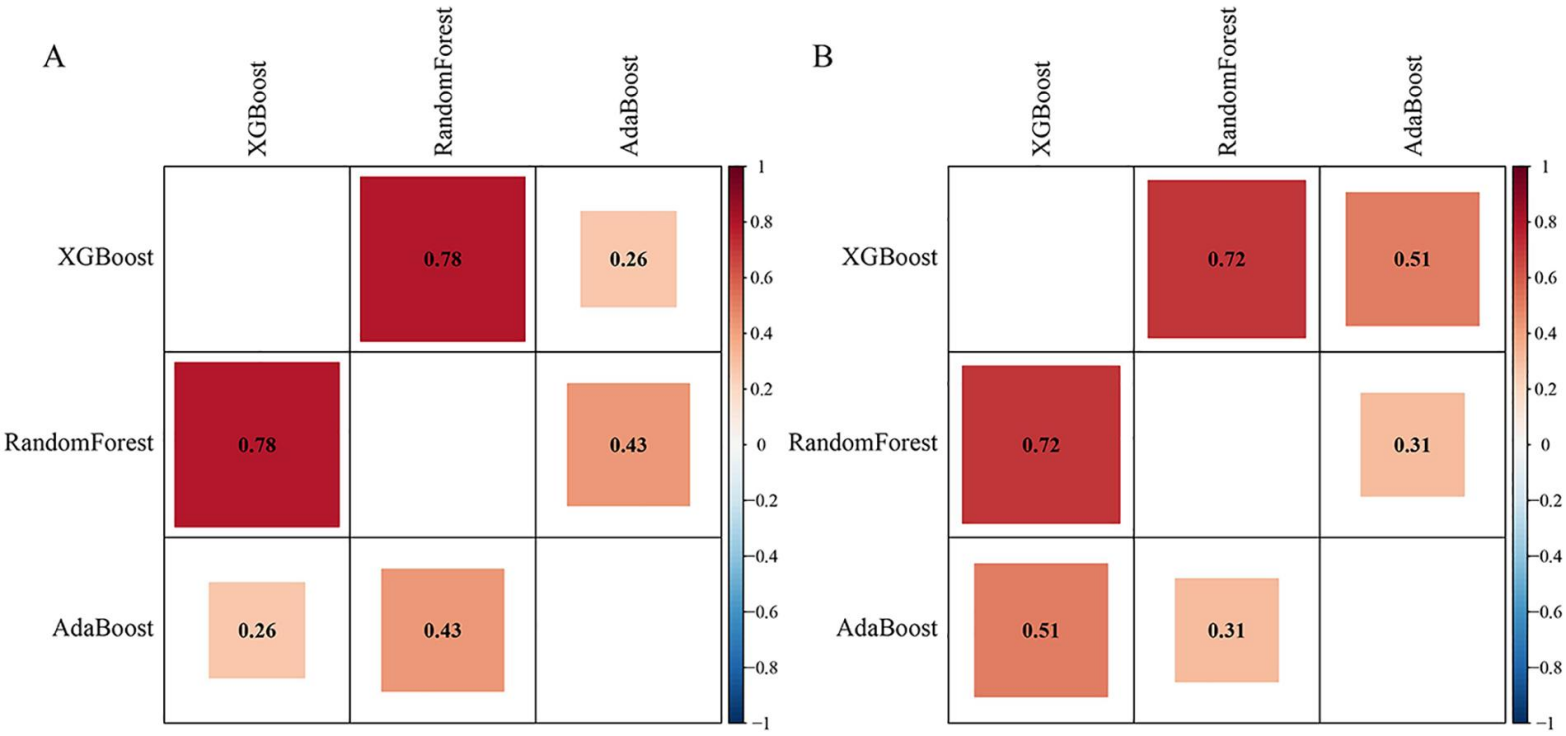
Statistical comparison of ensemble machine learning models using deLong test and IDI analysis. **(A)** presents the results of the DeLong test, and **(B)** displays the outcomes of the IDI analysis.

Pairwise AUC comparisons using the DeLong test showed no statistically significant differences among XGBoost, Random Forest, and AdaBoost. IDI analyses revealed modest increases in reclassification indices for XGBoost relative to AdaBoost (IDI = 0.042, *p* = 0.306) and Random Forest (IDI = 0.028, *p* = 0.511), as well as for Random Forest relative to AdaBoost (IDI = 0.014, *p* = 0.716), none of which reached statistical significance (Figure 5).

### 3.5 Feature importance analysis

**Figure 6:**
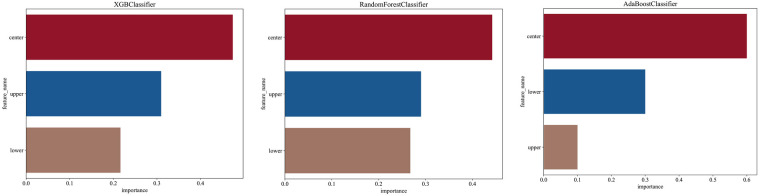
Feature importance analysis for the ensemble models.

Feature importance analysis was performed to assess the relative contribution of each MRI plane to the final diagnostic prediction. For all ensemble models, the central slice (the maximal ROI cross-section) was consistently identified as the most influential feature, contributing an average 50% across classifiers. The superior and inferior adjacent slices contributed 23% and 26%, respectively. Table 3 and Figure 6 display the detailed feature importance rankings across the three ensemble models.

**Table 3:** Relative feature importance of MRI slices in ensemble models.

Slice	Feature importance		XGBoost	Mean value
	AdaBoost	Random forest		
Center	0.60	0.44	0.47	0.5
Upper	0.10	0.29	0.31	0.23
Lower	0.30	0.27	0.22	0.26

## 4 Discussion

Currently, the diagnosis of SLAP lesions remains controversial in shoulder surgery, and there is a lack of effective, non-invasive, and accurate intelligent diagnostic methods in clinical practice (35, 36). In this study, we developed and validated a novel SLAP lesion diagnostic model utilising a 2.5D deep learning framework combined with ensemble learning. Comprehensive analysis of multi-slice MRI features yielded strong diagnostic metrics on the test set, with the XGBoost-based model achieving an AUC of 0.754 and a sensitivity of 0.933. Notably, these results compare favourably with previous MRI-based diagnostic studies. For example, the meta-analysis by Symanski et al. (8) reported an average sensitivity of 63% for SLAP diagnosis using conventional MRI, whereas our approach achieved a significantly higher sensitivity of 93.3%. The ensemble learning model based on deep learning-derived predictions demonstrated superior stability and generalisability. We also conducted a systematic comparison of three distinct ensemble algorithms and presented the decision-making mechanisms through visualised feature-importance analysis. These findings further substantiate the applicability of deep learning models in the diagnosis of SLAP lesions.

While imaging remains pivotal for the pre-operative identification of SLAP lesions, traditional radiological interpretation is limited by subjectivity and qualitative reporting, leaving valuable quantitative imaging information underutilized (37, 38). The principal advance of deep learning technology lies in its ability to automatically extract high-dimensional, discriminative features through hierarchical, non-linear transformations of imaging data (39, 40). As Gillies et al. (11) noted, “images are more than pictures, they are data.” This technique enables the detection of subtle and complex imaging patterns, thereby overcoming the limitations of manual feature engineering. The Wide_Resnet101_2 network employed in this study provides several key advantages by automatically extracting highly discriminative features directly from raw MRI data while minimizing human intervention, and its end-to-end mapping from image pixels to diagnostic predictions substantially improves the objectivity and reproducibility of model outputs. Furthermore, its superior non-linear modeling capacity enables the identification of complex imaging patterns and subtle morphological variations of SLAP lesions that would likely be missed by traditional assessment. Collectively, these strengths demonstrate the clinical potential of deep learning to enhance diagnostic accuracy in musculoskeletal imaging. Recent studies confirm the clinical value of deep learning in diagnosing shoulder disorders (41, 42). For example, Ni et al. (43) developed a multi-task deep learning system that achieved excellent performance in classifying supraspinatus tendon injuries. The classification accuracy of their system was significantly higher than that of radiologists on both intra-group and inter-group datasets (*p* < 0.001).

Ni et al. (44) made a significant contribution to the field by developing and externally validating the large-scale SLAP-Net model based on MRA data from 636 patients, all confirmed by arthroscopy. The model, trained on 514 cases and evaluated on an independent set of 122 patients from various MRI scanners, achieved excellent diagnostic performance (AUC = 0.92, accuracy = 0.85). Its performance was comparable to that of senior radiologists (*p* = 0.055) and significantly surpassed that of less experienced radiologists (*p* = 0.025). Nevertheless, because MRA requires intra-articular contrast injection, there is an increased risk of joint infection and associated complications, limiting its routine clinical use. Our study addresses this limitation by advancing a deep learning-based diagnostic approach for SLAP lesions using conventional MRI sequences as a non-invasive alternative. By relying solely on standard MRI protocols and eliminating the need for contrast agents, our method substantially reduces procedural risk and patient burden. In the study conducted by Ni et al., a 2.5D modelling strategy was utilised. The model was trained using a combination of axial and oblique coronal fat-saturation T1-weighted fast spin-echo (T1-FSE-FS) images that included the ROIs as input. The patient's final diagnosis was determined by the most frequently predicted category. Our approach employs a 2.5D architecture that is specifically designed to balance diagnostic accuracy, computational efficiency, and clinical practicality. Unlike traditional 2D single-plane analysis, our method integrates the maximal lesion slice with its adjacent sections, thereby fully exploiting the spatial distribution and three-dimensional morphology of SLAP lesions. Feature-importance analysis further supports the value of this approach: the central slice accounts for 50% of predictive information, whereas the contiguous upper and lower slices contribute 23% and 26%, respectively. The predominance of the central slice is plausible because it was defined as the slice with the largest ROI cross-sectional area, which is most likely to depict the lesion epicenter and the most discriminative changes at the superior labrum–biceps anchor complex. This is also consistent with routine clinical practice, where radiologists and surgeons typically prioritise the slice demonstrating the most conspicuous abnormality while using adjacent slices as complementary context. Compared with full 3D analyses, our 2.5D strategy confers notable advantages for real-world deployment: (1) markedly improved computational efficiency with significantly reduced training and inference times; (2) lower hardware demands, facilitating broader adoption across diverse healthcare environments; and (3) avoidance of excessive model complexity, enhancing scalability and practicality without compromising diagnostic performance.

Lin et al. (45) introduced a deep learning framework for the detection and classification of rotator cuff tears on shoulder MRI, distinguishing between no tear, partial-thickness tear, and full-thickness tear. Their architecture comprised four parallel 3D ResNet50 networks, trained via transfer learning. The final diagnostic classification was determined by averaging the predicted probabilities from each network and selecting the class with the highest mean probability. Notably, the study demonstrated that a multi-sequence input strategy significantly outperformed single-sequence input for identifying infraspinatus and subscapularis tendon tears, achieving AUCs of 0.89 and 0.90, respectively. Leveraging ensemble learning, our study introduces an innovative decision-level fusion strategy specifically designed for SLAP lesion diagnosis. Our model features a two-stage design: deep learning-based feature extraction followed by machine learning ensemble classification. In the first stage, the deep learning component automatically extracts high-level semantic features from multi-plane MRI images. In the second stage, an ensemble learning algorithm integrates the predictive probabilities from each imaging plane to generate the final diagnostic output. This framework ensures robust diagnostic performance and substantially enhances the interpretability of the model. Compared with traditional feature-level fusion methods, our decision-level fusion approach offers three distinct advantages: (1) it maintains the independent predictive value of each imaging plane; (2) it manages uncertainty more effectively by aggregating probabilistic outputs across views; and (3) the ensemble learning component provides greater transparency and interpretability than end-to-end deep learning, facilitating clinical acceptance and understanding of the model's decision-making rationale.

The three ensemble learning algorithms assessed in this study each demonstrated unique strengths. XGBoost achieved the highest overall diagnostic performance (AUC = 0.754, sensitivity = 0.933), likely due to its advanced gradient boosting framework and integrated regularisation, which effectively mitigate overfitting and enhance model generalisation. Random Forest exhibited strong fitting capability on the training set (AUC = 0.977) and maintained robust, balanced performance on the test set, attributable to its random sampling and feature selection strategies. AdaBoost also delivered reliable diagnostic accuracy in external validation. Importantly, DeLong's test indicated no statistically significant differences in AUC between the three models (all *p* > 0.05), highlighting their statistical equivalence. This convergence underscores the robustness of the deep learning–based feature extraction; different ensemble classifiers produced similarly strong results, enhancing both the credibility and translational potential of this approach.

To further investigate model interpretability, we employed Grad-CAM visualisation (46, 47), which demonstrated that the deep learning model predominantly attended to the biceps anchor region of the superior labrum—precisely the anatomical site implicated in SLAP lesions. This interpretability not only corroborates the clinical plausibility of the AI's decisions but also provides clinicians with tangible, visual diagnostic reference points. Feature-importance analysis of the ensemble machine learning model further elucidated its underlying decision process, revealing that the higher contribution from the central slice reflects the critical role of the core lesion region.

This study has several limitations. Key limitations of this study include its retrospective single-centre design, modest sample size, and lack of external validation, which may introduce selection bias and collectively limit the generalisability of the findings. In addition, although data augmentation was used to improve data diversity, it may introduce unrealistic transformations that could influence the interpretation of results. Future research should aim to expand the cohort size and include multi-centre, prospective external validation to better assess robustness and applicability. Second, our classification was limited to a binary SLAP lesion diagnosis, without finer subclassification by lesion type or severity, thereby restricting clinical utility and diagnostic granularity. Performance discrepancies between training and test sets across all models also suggest potential for further optimisation, particularly in handling complex cases. Third, the model was developed using imaging features alone, without incorporating clinical variables such as detailed physical examination findings or patient history. Incorporating multimodal data in future studies could improve diagnostic accuracy and real-world applicability. Finally, despite the use of Grad-CAM for visual interpretability, the complex “black-box” nature of deep learning remains a significant barrier to clinical adoption. Continued development of more transparent and user-friendly interpretability methods and visualisation tools is needed to strengthen clinician confidence and facilitate broader integration into clinical workflows.

## 5 Conclusion

In this study, the proposed model demonstrated favourable diagnostic performance for SLAP lesion detection on conventional shoulder MRI. Among the ensemble strategies, the XGBoost classifier achieved an AUC of 0.754 and a sensitivity of 0.933, supporting its potential value as a clinical decision-support tool. The multi-slice fusion strategy consistently highlighted the central slice as the most informative, with adjacent slices providing complementary contributions. Moreover, Grad-CAM visualisations showed anatomically consistent attention to the expected lesion region, further enhancing clinical interpretability and supporting the potential of this approach as a practical AI-assisted solution for diagnosing shoulder labral injuries.

## Data Availability

The raw data supporting the conclusions of this article will be made available by the authors, without undue reservation.
