# Correction: Diagnosis of SLAP lesions on shoulder MRI using a 2.5D deep learning and ensemble learning framework

**DOI:** 10.3389/fsurg.2026.1866165

**Published:** 2026-06-17

**Authors:** Hongyu Wang, Qingyun Xue, Lei Shi, Fei Wang, Guanghan Gao, Lin Wang

**Affiliations:** 1 Department of Orthopaedics, Beijing Hospital, National Center of Gerontology, Institute of Geriatric Medicine, Chinese Academy of Medical Sciences, Beijing, China; 2 Peking University Fifth School of Clinical Medicine, Beijing, China

**Keywords:** artificial intelligence, deep learning, diagnostic model, ensemble learning, shoulder MRI, SLAP lesion

There was an error in the description of the MRI sequences used in the Materials and methods section. The sequences were incompletely described as only “oblique coronal T2-weighted fat-suppressed sequence,” whereas both oblique coronal proton density-weighted fat-suppressed (PD FS) and oblique coronal T2-weighted fat-suppressed (T2WI FS) sequences were included.

A correction has been made to the section 2 Materials and methods, *2.3 MRI protocol and arthroscopic reference standard*, Paragraph Number 1:

“All patients underwent standardised non-contrast shoulder MRI on one of two 3.0 T MRI scanners. The standardised scanning protocol comprised multiple sequences. In this study, images from the oblique coronal proton density-weighted fat-suppressed (PD FS) and oblique coronal T2-weighted fat-suppressed (T2WI FS) sequence were analysed. Images were acquired with standardised soft-tissue window settings and exported in Digital Imaging and Communications in Medicine (DICOM) format. All arthroscopic procedures were performed by experienced orthopaedic surgeons. The intra-articular structures, including the glenohumeral joint, the long head of the biceps tendon, and the biceps–labral complex, were systematically examined through standard posterior, anterior, and anterolateral portals. The integrity, continuity, and glenoid attachment of the superior labrum were carefully assessed. A definitive diagnosis of SLAP lesion was made intraoperatively if a superior labral tear or detachment of the biceps anchor from the glenoid was observed. Rotator cuff pathology was also documented and categorised as no tear, partial-thickness tear, or full-thickness tear.”

A correction has been made to section 2 Materials and methods, 2.4 Image preprocessing and region of interest (ROI) annotation, Paragraph Number 1:

“The ITK-SNAP software (28) was used for manual delineation of the superior labrum on oblique coronal fat-suppressed sequences, including PD FS and T2WI FS.”

The original version of this article has been updated.

